# Arthrodesis of distal interphalangeal and thumb interphalangeal joint: a retrospective cohort study of 149 cases

**DOI:** 10.1186/s12891-024-07361-w

**Published:** 2024-04-02

**Authors:** Philip Silvano, Evelina Pantzar-Castilla, Eva Lundqvist

**Affiliations:** 1 Faculty of Medicine and Health, Örebro University, Örebro, Sweden; 2 Department of Orthopedics and Hand Surgery, University Hospital Örebro, Södra Grev Rosengatan, Örebro, 70185 Sweden

**Keywords:** Arthrodesis, Distal interphalangeal (DIP) joint, Thumb interphalangeal (IP) joint, Complications, Outcomes, Osteoarthritis, Arthritis

## Abstract

**Background:**

Arthrodesis of finger joints is often the last line of treatment of severe pain due to osteoarthritis, rheumatoid arthritis, or mallet finger. At the Department of Orthopedic and Hand Surgery, Örebro University Hospital (ÖUH) in Sweden, the Kirschner-wire technique was standard until 2020, when the headless compression screw technique was introduced as a complement. There is no consensus on which method is superior. The purpose of this study was to examine the outcomes and complications associated with distal interphalangeal (DIP) joint and thumb interphalangeal (IP) joint arthrodesis, and to see whether these correlated with patient-dependent and treatment-related factors.

**Methods:**

In a retrospective cohort study, we evaluated a total of 149 consecutive arthrodeses (118 DIP joint and 31 thumb IP joint) performed between 2012 and 2022. The primary outcome was risk factors for complications after arthrodesis.

**Results:**

Osteoarthritis was the most common indication (56%) for arthrodesis. The majority of the patients were females (74%), and the median age was 62 (range 18–86). The complication frequency was 35%, with infection being the most common (25%). Time to completed follow up was < 12 weeks in the majority of the cases (58%). There were no significant differences in complication rate between the 136 joints operated using Kirschner wire and the 13 joints operated using headless compression screws. There was no significant increased risk of complications among smokers or patients with rheumatoid arthritis. Diabetes and surgeon experience had a significant influence on the risk of complication (*p* = 0.036 and *p* = 0.006, respectively).

**Conclusions:**

Osteoarthritis was the most common indication for arthrodesis and postoperative complications occurred at a rate similar to that reported in the existing literature. Diabetes and surgeon experience were identified as factors increasing the risk of postoperative complications in these DIP/thumb IP joint arthrodeses. However, there was no significant difference between the two techniques (Kirschner wire and headless compression screws) regarding complications. Further studies are needed in order to determine the optimal type of operation and choice of implant.

**Trial registration:**

Researchweb CRIS #280,998, 26th of July 2023.

## Background

Pain, deformity, and instability are the indications for arthrodesis of the distal interphalangeal (DIP) finger joint and thumb interphalangeal (IP) joint. The causes of these above mentioned indications, possibly leading to arthrodesis, includes primary osteoarthritis, acute trauma, post-traumatic condition, and rheumatoid joint deformity [1]. Osteoarthritis is the most common reasons for DIP joint arthrodesis. At least 60% of individuals older than 60 years have radiographic DIP joint arthritis [2]. However, not all patients with radiographic osteoarthritis experience pain [3]. The primary treatment is conservative, and aimed at symptom relief using analgesics [4]. If this is not sufficient, surgical treatment with arthrodesis is indicated to relieve pain, correct deformity, and stabilize a unstable joint [1].

Multiple surgical techniques have been developed. The three most common fixation techniques in DIP and thumb IP joint arthrodesis are Kirchner wire (K-wire), headless compression screw, and K-wire with interosseous cerclage wire [1]. There is no consensus on which method should be used [5–8]. A retrospective study including 173 DIP joint and thumb IP joint arthrodeses concluded that arthrodesis of these joints is often associated with complications [9], reporting an overall complication frequency of 44% (minor complications 29%, major complications 15%). Minor complications were defined as prolonged pain (8%), dysesthesia (14%), and wound healing problems (7%), and major complications as osteomyelitis (15%), nonunion (6%), implant failure (4%), and malalignment (1%). The study further implies that fixation with a headless compression screw has been associated with slightly higher bone union rates, but increases the risk of certain minor complications that, however, do not affect the outcome. Moreover, the choice of surgical technique can affect the outcome, as headless compressions screw arthrodesis was associated with a significantly lower rate of major complications when compared with K-wire/cerclage [9]. A systematic literature review of 32 studies covering 1125 cases of DIP joint arthrodeses reported overall complication frequencies of 19% for headless compression screws and 13% for K-wires, while the results on bone union frequency ranged from 91 to 96% for the different methods [5].

Several studies have highlighted the significant variation in complication rate regarding the different methods [9, 10]. It is therefore important to have a standardized definition of complications and their documentation in order to avoid systematic error. All methods can lead to non-union, hardware migration, loosening, skin necrosis, permanent stiffness of the proximal interphalangeal (PIP) joint, paresthesia, and osteomyelitis [11]. Previous studies of the headless compression screw imply that it is a reliable option for small joint arthrodesis [6, 10, 12]. Screw fixation is a widely- accepted method, but complications do exist; in particular migration of the screw head leading to decreased compression of the joint surfaces or protrusion of the screw head in the fingertip necessitating removal [10].

Patient-related factors can affect the risk of major complications. Smokers have a greater risk than non-smokers [9, 13]. Diabetes [14, 15] and rheumatoid arthritis [16, 17] also increase the risk of complications and affect osseous healing negatively. A retrospective study of 310 arthrodeses found that finger DIP and thumb IP joint arthrodesis generally resulted in favorable outcome in terms of bone union, regardless of the underlying medical condition or technical details of the surgical operation [1]. However, the authors emphasized the importance of surgical skill in order to decrease bone non-union and complications. Another retrospective study showed that in comparison to primary surgery, revisional arthrodesis was five times more frequently associated with major complications [9]. A previous study of the headless compression screw in DIP joint arthrodesis showed that union within 3 months occurred in 89% of cases, delayed union (3–6 months) occurred in 6%, and nonunion requiring subsequent revision arthrodesis occurred in 5% [8].

The purpose of DIP and IP joint arthrodesis is a painless and stable joint. At the Department of Orthopedic and Hand Surgery, Örebro University Hospital (ÖUH) in Sweden, the K-wire adaptation technique was standard until 2020, when the headless compression screw was introduced as a complement. The aim of this study was to investigate the outcomes and complications in a retrospective cohort study of 149 cases of DIP/thumb IP joint arthrodesis in patients operated at our department, and the correlation with patient-dependent and treatment-related factors.

## Methods

### Study design

The study was approved by the Swedish Ethical Review Authority (reference number 2023-04706-01).

In this retrospective cohort study, all patients operated with DIP joint or thumb IP joint arthrodesis, at the Department of Orthopedic and Hand Surgery, ÖUH, between January 1, 2012 and December 31, 2022 were identified using the ICD-10 procedure code NDG46. A total of 244 DIP, IP, and PIP joint arthrodeses were performed at the department during this period. Inclusion criteria was patients operated with DIP joint or thumb IP joint arthrodesis with ICD-10 procedure code NDG46. Exclusion criteria included patients under the age of 18 years and revisional arthrodesis. In patients who had more than one arthrodesis in the same session, each arthrodesis was considered as a separate case. A total of 149 cases (118 finger DIP and 31 thumb IP joint) met the inclusion criteria and were available for analysis.

### Data collection

Patient records were analyzed September 2023, covering data on age, sex, indication for arthrodesis, digit operated, comorbidity, smoking habits, surgical technique, type of anesthesia, operation time, surgeon experience, time to osseous union, number of doctor’s visits, time of immobilization, and complications. Time to completed follow-up and duration of immobilization were counted in number of weeks and rounded to the nearest integer.

### Definitions

In this study, a complication was defined as any deviation from the ideal postoperative healing process. Prolonged pain lasting more than three months after surgery was defined as a complication [18]. Infection was considered as a complication when the doctor reported clinical signs of infection and prescribed antibiotics. No verified bacterial culture was needed. If the infection developed to osteitis/osteomyelitis, this was considered a complication of its own. All patients were encouraged to stop smoking preoperatively. Smokers were defined as those who smoked at the time of surgery or at the first follow-up visit.

### Surgical techniques

All surgeries were performed with a cuff on the forearm or finger. A H- or Y-shaped incision was made dorsally over the DIP/thumb IP joint. The extensor tendon was then exposed and divided obliquely. Both collateral ligaments were excised so that the joint could be flapped up. The joint surfaces (cartilage and subcortical bone) was recessed with a sagittal saw and osteophytes were recessed with Gouge forceps. The next step was adaptation of the arthrodesis position with either K-wires or a headless compression screw.

### K-wire

Two K-wires (0.8–1.2 mm) were positioned into the distal phalanx. At least one of the two K-wires was inserted oblique with inside-out technique. The other K-wire was either longitudinal or oblique. Fluoroscopy was used to verify the K-wire position. The K-wires were either left outside the skin or pinched at cortical level and left in place. The extensor tendon was sutured with 4 − 0 Vicryl and the skin with 4 − 0 Ethilon.

### Acutrak 2 headless compression screw

Acutrak 2 Headless compression screw (Acumed, Oregon, USA) was used for the screw technique. Screw size varied upon the individual patient and appropriate size was decided by the operating surgeon. First, a guidewire was inserted into the distal phalanx in a proximal to distal direction, emerging through the fingertip. The middle and distal phalanges were then adjusted, and the guidewire was advanced proximally into the middle phalanx under fluoroscopic control. After pre-drilling and placing the screw from distal to proximal over the guidewire, the guidewire was removed. The extensor tendon was sutured with 4 − 0 Vicryl and the skin with 4 − 0 Ethilon.

### Postoperative treatment

Postoperative treatment began with two weeks with a cast which was then changed to an individually-adapted orthosis provided by an occupational therapy practitioner. The sutures were removed at the same visit. Six to eight weeks postoperatively the patient had a first follow-up visit to the surgeon with a radiological assessment and clinical evaluation. K-wires were usually extracted after 8–10 weeks if there was no complication and the joint seemed clinically and/or radiologically healed. All patients were seen by an experienced hand therapist in order to mobilize the finger.

### Statistical analysis

IBM SPSS Statistics (IBM SPSS Statistics for Windows, Version 28.0.) was used for statistical analyses. Groups were compared using the Mann-Whitney U-test, chi^2^ tests, and binary logistic regression. Mann-Whitney U-test was used for group comparisons of non-normally distributed data, otherwise chi^2^ test was used. Binary logistic regression was used for analyzing the effect of a continuous variable on a binary outcome. *p* < 0.05 was considered to be statistically significant. Median (range) was used to summarize the data.

## Results

### Demographics

Demographics are demonstrated in Tables 1 and 2. A total of 149 arthrodeses of DIP joint (79%) and thumb IP joint (21%) were conducted during 2012–2022. The majority of the patients were females (74%), and the median age was 62 (range 18–86). Osteoarthritis was the most common indication (56%), followed by arthritis (15%). The index finger was the most frequently operated digit.

**Table 1:** Characteristics of the study population

Total arthrodesis (n)	149	
**Sex (n, %)**		
Female	111	74.5
Male	38	25.5
**Age at time of surgery (median, range)**	62	18–86
**Digit (n, %)**		
Index finger	48	32.2
Middle finger	38	25.5
Thumb	31	20.9
Ring finger	16	10.7
Small finger	16	10.7
**Comorbidities (n, %)**		
Rheumatoid arthritis	29	19.5
Diabetes	22	14.8
Smoking	9	6.0
**Indication for arthrodesis (n, %)**		
Osteoarthritis	83	55.7
Arthritis	23	15.4
Post-trauma	16	10.7
Mallet finger	14	9.4
Acute trauma	9	6.0
Flexor pollicis longus injury	2	1.3
Secondary arthritis	2	1.3

**Table 2:** Characteristics of the arthrodeses

Total arthrodesis (n)	149	
**Technique (n, %)**		
K-wire	136	91.3
* Buried subcutaneous*	88	64.7
*Left outside skin*	48	35.3
Headless compression screw	13	8.7
**Complications (n, %)**		
No complications	97	65.1
Infection	37	25.0
Pain	17	11.4
Revisional arthrodesis	8	5.4
Nonunion	7	4.7
Amputation	2	1.3
Early removal of K-wire	2	1.3
Malalignment	2	1.3
Osteitis/osteomyelitis	2	1.3
Pseudoarthritis	2	1.3
Loosening of K-wire	1	0.7
Loss of function	1	0.7
**Anesthetic method** **(n, %)**		
Local anesthesia	125	83.9
General anesthesia	24	16.1
**Operation time in minutes (mean, range)**		
K-wire	55	16–130
Headless compression screw	41	22–70
**Surgeon experience (n, %)**		
Board certified specialist	76	51.0
Senior specialist	55	36.9
Resident	18	12.1
**Duration of immobilization in weeks (median, range)**		
K-wire	9	2-107
Headless compression screw	8	6–20
**Time to completed follow-up (n, %)**		
< 12 weeks	87	58.4
12–24 weeks	39	26.2
> 24 weeks	23	15.4
**Number of visits to doctor (median, range)**	3	1–22
**Ascertained healing (n, %)**		
Clinical	125	83.9
Radiological and clinical	23	15.4
Radiological	1	0.7

### Outcome, complications and univariate analysis

**Fig. 1:**
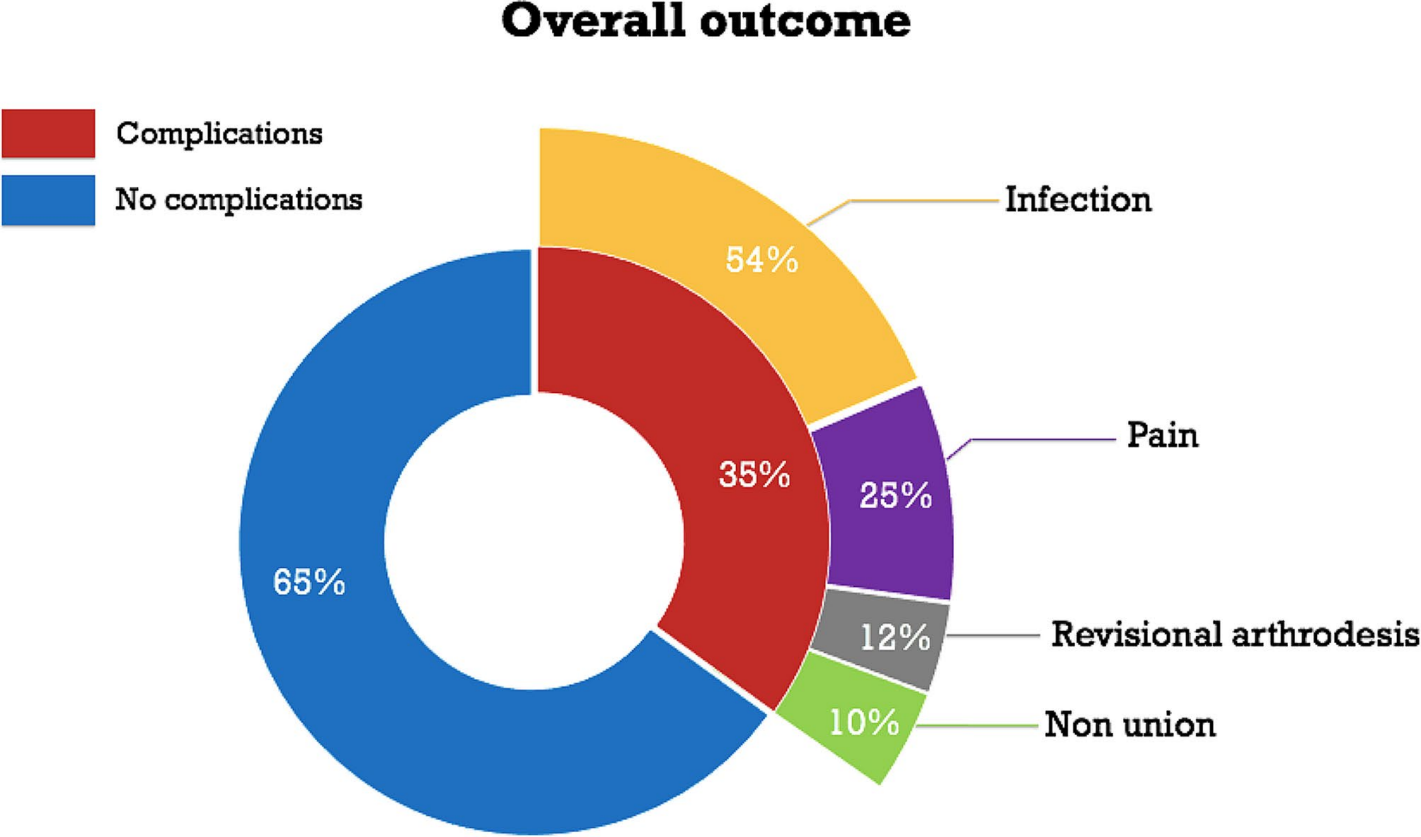
Complication rate for DIP/thumb IP joint arthrodesis (*n* = 149). Complications with *n* < 3 are not included in the breakdown

The median range of immobilization was 9 weeks when using K-wire (range 2-107) and 8 weeks when using headless compression screw (range 6–20). Time to completed follow up was < 12 weeks in the majority of the cases (58%) and healing was ascertained clinically in 84% of the cases (Table 2). The median number of visits to the doctor was 3 (Table 2). The complication frequency was 35%, with infection being the most common (25%) (Fig. 1). K-wire fixation was the most frequently used technique (91%). There were no significant differences in complication rate between the 136 joints operated using K-wire and the 13 joints operated using headless compression screws (*p* = 0.769; Table 3). There was no significant increased risk of complications for smokers or patients with rheumatoid arthritis. The median operation time was 51 min (SD = 71.2) for the complication group and 47 min (SD = 74.8) for the no complication group. When using logistic regression for all arthrodesis, it showed that longer operation time is not a risk factor for complication (*p* = 0.7) (Table 3). Diabetes and surgeon experience had a significant influence on the risk of complication (*p* = 0.036 and *p* = 0.006, respectively) (Tables 3 and 4).

**Table 3:** Incidence of complications in potential risk groups after DIP/thumb IP joint arthrodesis

Potential risk factor	Total, n	Complication, n (%)	p-value
Board certified specialist	76	26 (34)	**0.006** ^**a**^
Senior specialist	55	14 (25)	
Resident	18	12 (67)	
No diabetes	127	40 (31)	**0.036** ^**a**^
Diabetes	22	12 (55)	
Non-smoker	140	47 (34)	0.278^b^
Smoker	9	5 (56)	
No rheumatoid arthritis	120	44 (37)	0.357^a^
Rheumatoid arthritis	29	8 (28)	
K-wire	136	47 (35)	0.769^b^
Headless compression screw	13	5 (38)	
K-wire buried subcutaneous	88	27 (31)	0.198^a^
K-wire left outside skin	48	20 (42)	
DIP joint	118	44 (37)	0.233^a^
Thumb IP joint	31	8 (26)	
Local anesthesia	125	44 (35)	0.861^a^
General anesthesia	24	8 (33)	
Operation time in minutes	147	51 (35)	0.746^c^

p-values in bold indicate statistical significance (*p* < 0.05)

^a^ Chi^2^ test

^b^ Fisher’s exact test, used when *n* < 8

^c^ Logistic regression with all arthrodesis included (missing data *n* = 2)

**Table 4:** Comparison of surgeon experience regarding complications

Surgeon experience	Surgeon experience	p-value
Resident	Senior specialist	**0.003**
Resident	Board certified specialist	**0.028**
Board certified specialist	Senior specialist	0.606

p-values in bold indicate statistical significance (*p* < 0.05) measured with a chi^2^ test

## Discussion

Our study revealed a complication frequency of 35% after DIP/thumb IP joint arthrodesis. This can be compared with 44% complication rate in 173 cases reported by Runkel et al. [9]. In our study, only 13 arthrodeses were performed using a headless compression screw. This was a much smaller number than expected, making it difficult to compare with the 136 arthrodeses using K-wire adaptation. This is a limitation in our study. However, the complication rates for headless compression screw technique and K-wire adaptation were 38% and 35% respectively. Despite the small number of cases in the headless compression screw group, the results indicate no difference in complication rate between the two techniques. The trend is similar to other studies; the two techniques appear to be comparable regarding the risk of postoperative complications [5]. A systematic literature review including 1125 cases reported overall complication frequencies of 19% for headless compression screw and 13% for K-wire [5]. However, the authors warned that the results should be interpreted with caution due to the level of evidence of the studies and the heterogeneous mix of patients in terms of age and indications for arthrodesis.

The intention at our department has been to use headless compression screws. However, the typical patient is a relatively elderly woman with thin finger bones, leading to the screw being perceived as too large. Moreover, this population is more susceptible to injury since their bones are more osteoporotic [19]. Earlier studies have shown that attention to the size of the distal phalanx is important to avoid complications in smaller fingers [20]. The headless compression screw technique was introduced at our department in 2020 as a complement to K-wire, and as always with the implementation of new methods, there has been a learning curve, meaning that the use of this technique may increase in the future.

Union within 3 months occurred in 58% and delayed union (12–24 weeks) in 26% of the 149 cases in our study. Kocak et al. investigated 51 patients operated with a headless compression screw reported union within 3 months in 89% and delayed union (3–6 months) in 6% of cases [8]. This difference in rates could be due to the use of different surgical technique, since the majority of cases in our study were operated using K-wire. The financial perspective can also be considered. A systematic review concluded that the headless compression screw can facilitate earlier mobilization and potentially earlier return to work when compared to the K-wires, and so although the cost of using a screw is significantly greater in comparison with a K-wire, the potential savings may compensate for the increased cost [5].

Infection and pain were the most common complications in this study. It should be emphasized that several cases involved more than one complication; for example, both infection and pain. Additionally, there was substantial variation in the degree of infection; some improved immediately after the patient received antibiotics, while others led to osteitis and protracted problems. Thus, when reading the results, it is important to keep in mind that the number of complications does not necessarily reflect the degree of difficulty it implies.

If the K-wires caused no inconvenience and were clinically stable at the first follow-up visit after 6–8 weeks, the doctor often decided to leave the wires in place for additional weeks in order to be completely sure of bone union in cases of remaining radiographic joint space. These additional weeks delayed the time to “completed follow-up” according to our way of calculating bone union. The benefit of headless compression screw in comparison with K-wiring is that the compression of the joint space leads to faster union [5]. It is difficult to define what a healed arthrodesis means in concrete terms. Radiological and clinical findings may not correspond, and patients may have different levels of acceptance regarding postoperative stability, function, and pain in the joint. However, in our study, healing was ascertained clinically in 84% of cases. This was a retrospective study with no standardized follow-up: The period to completion of follow-up visits was used as a measurement of the point when both doctor and patient were satisfied with the result. In future studies it might be preferable to include both radiological and clinical follow-up.

A study reporting on a retrospective series of 310 cases determined that the surgeon’s lack of specialty training was the strongest predictor of non-union, increasing the risk almost fourfold [1]. This is in line with our finding, that lower surgeon experience was significant correlated with higher complication incidence. The difference was seen between residents and board certified specialists, and was even stronger between residents and senior specialists. No statistical difference was shown between board certified specialists and senior specialists. To minimize the risk of postoperative complications it could be reasonable to adjust the resident training programs and increase the degree of specialist supervision during surgery.

Diabetes also increased the risk of postoperative complications in our study, as previously shown by Jiao et al. [13]. Rheumatoid arthritis, on the other hand, showed no statistically significantly increased risk of postoperative complications, which contradicts the results of Fowler et al. [15]. We were also not able to show that smoking was a risk factor for postoperative complications, which again is contrary to previous research [13, 21]. Since the prevalence of smoking in our study was 6%, which is a quite low, it is likely that the reason for this non-association was underreporting of smoking habits by the patients or lack of statistical power.

In this study, operation time and anesthetic method were analyzed as risk factors for complications when performing DIP/thumb IP joint arthrodesis. To the best of our knowledge, these factors have not been explored in the existing literature. The results showed that neither operation time nor type of anesthesia (local vs. general) had an impact on the complication rate.

Cases were identified via the ICD-10 procedure code NDG46, thus if other procedure codes were used or the surgery was incorrectly coded when performing DIP/thumb IP joint arthrodesis, there may have been a selection bias. Another limitation in this study is the lack of multivariate analysis, which could have amplified the results. This should be considered in future studies. Conversely, one strength of this study is the long timeframe of over a decade. In addition, there was no loss to follow-up, and so the results give a good picture of the techniques used and the complication rate at the center. Another limitation, mentioned and discussed above, was the low number of cases fixated with headless compression screw. Lack of standardized follow-up is a limitation mentioned above, due to the retrospective design of this study.

## Conclusion

Postoperative complications in our study occurred at a rate similar to that reported in existing literature [9]. Diabetes and surgeon experience were identified as factors increasing the risk of postoperative complications after DIP/thumb IP joint arthrodesis. However, there was no significant difference between the two techniques (K-wire and headless compression screw) regarding complications. Further studies are needed in order to determine the optimal type of operation and choice of implant.

## Data Availability

The datasets used and analyzed during the current study are available from the corresponding author on reasonable request.

## Abbreviations

DIP: distal interphalangeal
IP: interphalangeal
K: wire - Kirschner wire
PIP: proximal interphalangeal
ÖUH: Örebro University Hospital
